# Diffuse Axonal Injury: Epidemiology, Outcome and Associated Risk Factors

**DOI:** 10.3389/fneur.2016.00178

**Published:** 2016-10-20

**Authors:** Rita de Cássia Almeida Vieira, Wellingson Silva Paiva, Daniel Vieira de Oliveira, Manoel Jacobsen Teixeira, Almir Ferreira de Andrade, Regina Márcia Cardoso de Sousa

**Affiliations:** ^1^School of Nursing, University of São Paulo (USP-SP), São Paulo, Brazil; ^2^Department of Neurology, School of Medicine, University of São Paulo (USP-SP), São Paulo, Brazil

**Keywords:** head trauma, diffuse axonal injury, Glasgow Outcome Scale, recovery, severe traumatic brain injury, cohort study

## Abstract

Diffuse axonal injury (DAI), a type of traumatic injury, is known for its severe consequences. However, there are few studies describing the outcomes of DAI and the risk factors associated with it. This study aimed to describe the outcome for patients with a primary diagnosis of DAI 6 months after trauma and to identify sociodemographic and clinical factors associated with mortality and dependence at this time point. Seventy-eight patients with DAI were recruited from July 2013 to February 2014 in a prospective cohort study. Patient outcome was analyzed using the Extended Glasgow Outcome Scale (GOS-E) within 6 months of the traumatic injury. The mean Injury Severity Score was 35.0 (SD = 11.9), and the mean New Injury Severity Score (NISS) was 46.2 (SD = 15.9). Mild DAI was observed in 44.9% of the patients and severe DAI in 35.9%. Six months after trauma, 30.8% of the patients had died, and 45.1% had shown full recovery according to the GOS-E. In the logistic regression model, the severity variables – DAI with hypoxia, as measured by peripheral oxygen saturation, and hypotension with NISS value – had a statistically significant association with patient mortality; on the other hand, severity of DAI and length of hospital stay were the only significant predictors for dependence. Therefore, severity of DAI emerged as a risk factor for both mortality and dependence.

## Introduction

Diffuse axonal injury (DAI), the microscopic damage to the axons in the brain neural tracts, corpus callosum, and brainstem, is associated with significant mortality and morbidity. The occurrence of DAI depends on the mechanism of injury; it is more common in higher energy trauma, especially traffic accidents (1–3).

Diffuse axonal injury is clinically defined by coma lasting 6 h or more after traumatic brain injury (TBI), excluding cases of swelling or ischemic brain lesions (2). DAI is considered the most important factor in determining morbidity and mortality in victims of TBI and is the most common cause of posttraumatic coma, disability, and a persistent neurovegetative state (1, 2).

Diffuse axonal injury causes cognitive, physical, and behavioral changes that compromise social reintegration, return to productivity, and quality of life of patients and their families (1–10). These changes persist beyond the acute phase of treatment and continue for a long period after the traumatic event. Because the brain tissue is functionally impaired but not destroyed, the brain may gradually regain normal function as the clinical condition stabilizes and neural connections are remodeled due to plasticity (9–12).

According to some authors (7, 9, 10, 13), understanding the variables associated with recovery after TBI is needed for the development of individualized therapy, the evaluation of care provided, and the development of systematic care focused on patient rehabilitation. It is also important for the evaluation of the efficacy of new techniques and treatments, as these should result in better outcome and survival.

Diffuse axonal injury, and more generally TBI, often results in physical, cognitive, and behavioral impairments that can be temporary or permanent (1–10). Research on outcome after TBI, using scales of function and performance in activities of daily living (ADLs), depicts the individual and social consequences of these changes suffered by patients after TBI.

The outcome of patients after DAI has been linked to the number of lesions identified through imaging. A longitudinal study that analyzed the evolution of traumatic axonal injury using magnetic resonance imaging (MRI) of 58 patients with moderate or severe TBI showed that the greater the number of lesions observed early after trauma, the greater the impairment of functionality after 12 months (14). A study of 26 DAI patients (15) indicated that the volume and number of lesions identified by MRI performed within 48 h of hospital admission strongly correlated with the level of disability observed at the time of hospital discharge.

Few studies on DAI patients have focused on the clinical and sociodemographic factors associated with outcome and mortality. Thus, the aim of this study was to describe the outcome of patients with primary diagnosis of DAI and to identify clinical and sociodemographic factors associated with mortality and functional capacity 6 months after the injury.

## Materials and Methods

### Patients

This was a prospective cohort study, with data collected at the time of hospital admission and 6 months after DAI in the Neurosurgical Outpatient Clinic and Trauma at the Central Institute of the Hospital das Clínicas da Faculdade de Medicina da Universidade de São Paulo (IC/HCFMUSP) in São Paulo, Brazil.

Seventy-eight patients with DAI admitted to the IC/HCFMUSP from July 2013 to February 2014 were enrolled in the study. Patients eligible for the study had Glasgow Coma Scale (GCS) scores of ≤8 at admission, were between 18 and 60 years old, and had a computed tomography (CT) scan showing either normal outcome or signs of DAI. DAI was confirmed by signs of injury identified in CT or MRI scans by neurosurgeons experienced with this type of injury. Cases with GCS ≤8, and without MRI and normal CT were also diagnosed as DAI (16–21). The study criteria excluded patients who were admitted to these hospitals more than 6 h after trauma, who were transferred from other hospitals with previous diagnosis of TBI, or who had psychiatric disorders or other injuries to the head or spinal cord with a severity score ≥3, according to the Abbreviated Injury Scale (21).

For each patient, the following data were recorded:

- Sociodemographic characteristics: age, sex, marital status, race, and occupational status.
- Characteristics related to trauma and pre-hospital care (PHC): traffic accident, type of PHC, orotracheal intubation (OTI) at the scene, and alcohol intake.
- Characteristics related to admission: sedation, respiratory rate (RR), hypotension, bradycardia, tachycardia, hypoxia by peripheral oxygen saturation (SpO_2_), hypoxia by partial pressure of oxygen (PO_2_), hypoglycemia, hyperglycemia, and pupillary abnormalities.
- Characteristics related to hospital stay: intensive care unit (ICU) stay, use of continuous sedation, treatments with drugs that act on the central nervous system (CNS), surgery, second surgery, infection, other complications, early DAI signs in CT, intracranial pressure (ICP) monitoring, intracranial hypertension (ICH), hypotension, hypertension, hypothermia, hyperthermia, hypoglycemia, and hyperglycemia.

The secondary systemic injuries recorded for each patient during admission and hospital stay are as follows:

- RR: normal 10–29 breaths per minute and RR changes <10 or >29 breaths per minute.
- Hypotension: systolic blood pressure <90 mmHg;
- Hypertension: systolic blood pressure ≥160 mmHg;
- Bradycardia: heart rate variability <50 beats per minute;
- Tachycardia: heart rate variability >100 beats per minute;
- Hypoxia by SpO_2_: SpO_2_ <90%;
- Hypoxia by PO_2_: PO_2_ <60%;
- Hypoglycemia: glycemia <70 mg/dL;
- Hyperglycemia: glycemia >160 mg/dL;
- Hypothermia: axillary temperature ≤35°C;
- Hyperthermia: axillary temperature ≥38°C;
- Early DAI signs in CT: based on cranial CT scan in the first 72 h of hospital admission, this included individuals with intraventricular hemorrhage, subarachnoid hemorrhage, gliding contusion, or diffuse swelling with deletions of the basilar cisterns or grooves (indirect signs of injury) (16–21).

### Procedure

Sociodemographic data, clinical variables related to trauma, PHC, admission and hospitalization details, and variables related to the severity and consequences of DAI were collected for all of the patients. During the 6-month follow-up interview, sociodemographic data and data related to the traumatic event were confirmed, and information on the functional outcome of victims was recorded using the Extended Glasgow Outcome Scale (GOS-E).

Trauma severity was estimated with the Injury Severity Score (ISS) (22) and the New Injury Severity Score (NISS) (23). The Maximum Abbreviated Injury Scale (MAIS), referring to the head region (MAIS-Head) (21), was used to characterize the severity of TBI.

For DAI severity, Gennarelli’s clinical classification, rating diffuse lesions as mild, moderate, or severe, was applied (1, 2). In mild DAI, coma lasts 6–24 h. In moderate DAI, coma lasts longer than 24 h but without abnormal posturing. In severe cases of DAI, coma duration is longer than 24 h, and signs of brainstem impairment can also be observed (1, 2). Patients were considered to have awakened from the coma when they scored 6 on the best motor response (BMR) in the GCS.

To evaluate the functional outcome, GOS-E was applied, encompassing seven categories: upper good recovery, lower good recovery, upper moderate disability, lower moderate disability, upper severe disability, lower severe disability, and persistent vegetative state. For the patients alive at 6 months after trauma, level of dependence was determined according to the criteria of this scale; patients included in the categories of upper good recovery, lower good recovery, upper moderate disability, and lower moderate disability were grouped as independent, whereas those with upper severe disability, lower severe disability, or in a persistent vegetative state were classified as dependent (24, 25).

The study was approved by the Research Ethics Committee of the Escola de Enfermagem da Universidade de São Paulo and of the Escola de Medicina da Universidade de São Paulo (certificate of submission to ethics review number 14115513.1.3001.0068). All participants freely consented to participation and signed the informed consent form. Written and informed consent was given by all participants who were clinically able to do so; otherwise, the forms were signed by their legal representatives.

### Statistical Analysis

The information related to this investigation was stored in a computerized database in the Statistical Package for Social Sciences software version 17.0 (SPSS^®^, IBM).

To identify associations between the variables of interest and the outcomes of mortality and dependence 6 months after trauma, comparisons were made between groups of individuals who died or survived and between those who were dependent or independent, as determined by the GOS-E. In these comparisons, Pearson’s chi-square and Fisher’s exact tests were applied for categorical and numerical variables, respectively. Both discrete and continuous numerical variables were compared using Student’s *t*-test.

Multiple logistic regression analysis with stepwise forward method was performed on the variables associated with risk factors for mortality and dependence. Separate models were created with ISS and NISS for these variables, as ISS and NISS estimate the overall severity of the trauma and thus display multicollinearity problems. During modeling, the final model used only those variables that showed statistical significance in the logistic regression model (*p* ≤ 0.05).

## Results

Between July 2013 and February 2014, 78 patients with DAI admitted to IC/HCFMUSP met the inclusion criteria of the study and participated in the survey at the hospital admission stage. Of these patients, 24 (30.8%) died during the following 6 months, 51 (65.4%) were evaluated 6 months after DAI, and 3 (3.8%) withdrew from the study after hospital discharge.

Table 1 shows that the vast majority of the study participants were male (89.7%) and employed at the time of the injury (89.7%). The sample consisted mostly of young people between 18 and 28 years of age (43.6%), and the mean age of patients was 32 years (SD = 11.2). Participants who did not complete primary education only constituted 48.7% of the sample, and the average length of education was 9.1 years (SD = 9.1). Most of the trauma victims were white (65.4%) and single (51.3%); 73.0% had monthly per capita family income between one and five times the minimum wage, with an average income of R$1,290.98 (SD = R$2,282.64).

**Table 1:** **Comparisons between patient conditions (dead or alive, independent or dependent) at 6 months after diffuse axonal injury (DAI) in relation to sex, race, marital status, and occupational status at the time of trauma**.

Sociodemographic characteristics	Survival		*p*-Value	GOS-E		*p*-Value
	No *n* (%)	Yes *n* (%)		Independent *n* (%)	Dependent *n* (%)	
**Sex**						
Male	22 (91.7)	48 (88.9)	>0.999	40 (88.9)	5 (83.3)	0.548
Female	2 (8.3)	6 (11.1)		5 (11.1)	1 (16.7)	
**Marital status**						
Single	12 (50.0)	28 (51.9)	0.971	23 (51.1)	4 (66.7)	0.659
Married	10 (41.7)	21 (38.9)		18 (40.0)	2 (33.3)	
Separated	2 (8.3)	5 (9.2)		4 (8.9)	–	
**Race**						
White	16 (66.7)	35 (64.8)	0.874	29 (64.4)	4 (66.7)	>0.999
Black	8 (33.3)	19 (35.2)		16 (35.6)	2 (33.3)	
**Occupational status**						
Employed	20 (83.3)	50 (92.6)	0.242	42 (93.3)	6 (100)	>0.999
Unemployed	4 (16.7)	4 (7.4)		3 (6.7)	–	

*HCFMUSP, 2013–2014*.

*GOS-E, Extended Glasgow Outcome Scale*.

As shown in Table 2, the main cause of DAI in this study was traffic accidents, with motorcyclists being the largest group of trauma victims (43.6%) in those events, followed by car occupants (25.6%). A large proportion of the patients (42.3%) referred to alcohol intake in the period immediately preceding the trauma event. All participants were transported to the hospital by pre-hospital emergency care services, with a significant proportion of air transport use (47.4%) for the victims. Individuals in a coma (GCS ≤ 8) constituted 75.7% of trauma victims, with 79.5% of survey participants intubated at the scene of the incident.

**Table 2:** **Comparisons between patient conditions (dead or alive, independent or dependent) at 6 months after DAI in relation to characteristics related to trauma and pre-hospital care (PHC)**.

Characteristics related to trauma and PHC	Survival		*p*-Value	GOS-E		*p*-Value
	No *n* (%)	Yes *n* (%)		Independent^a^ *n* (%)	Dependent^a^ *n* (%)	
**Traffic accident**						
Yes	20 (83.3)	45 (83.3)	>0.999	36 (80.0)	6 (100.0)	0.575
No	4 (16.7)	9 (16.7)		9 (20.0)	–	
**Type of PHC**						
Air	11 (45.8)	26 (48.1)	0.850	22 (48.9)	2 (33.3)	0.671
Land	13 (54.2)	28 (51.9)		23 (51.1)	4 (66.7)	
**OTI at the scene**						
Yes	18 (75.0)	44 (83.0)	0.535	38 (86.4)	4 (66.7)	0.242
No	6 (25.0)	9 (17.0)		6 (13.6)	2 (33.3)	
**Alcohol intake**						
Yes	8 (33.3)	25 (46.3)	0.285	20 (44.4)	2 (33.3)	0.688
No	16 (66.7)	29 (53.7)		25 (55.6)	4 (66.7)	

*HCFMUSP, 2013–2014*.

*^a^Excludes 1 case without information*.

*OTI, orotrachial intubation*.

Regarding the severity of the trauma, ISS ranged from 17 to 75, with a mean of 35 (SD = 11.9) and median of 33. As for the distribution of the victims in the three categories of severity, there were no patients with mild trauma (ISS < 16), 19.2% with moderate trauma (≥16 and <25), and the majority had severe trauma (ISS ≥ 25). According to NISS, almost all of the victims (91.0%) had severe trauma (NISS ≥ 25). The average for this index was 46.2 (SD = 15.9), and the median was 43, ranging from 18 to 75.

All DAI patients had MAIS score ≥4 in the head region, with an average of 4.6 (SD = 0.5). Critical injuries, i.e., MAIS score = 5, were found in 55.1% of the sample. Mild DAI was observed in 44.9% of the victims, moderate DAI in 19.2%, and severe DAI in 35.9%.

The clinical conditions of the victims on arrival at IC/HCFMUSP are shown in Table 3. RR was altered in 10.3% of the sample, with tachypnea as the most frequent change (6.4%). Hypoxia was detected by SpO_2_ measurement in 15.3% (SpO_2_ < 90%) and by PO_2_ in 14.1% (PO_2_ < 60%) of the trauma victims. Of the study participants, 19.2% had an SBP <90 mmHg, 2.5% experienced cardiac arrest, and most (52.5%) had tachycardia at this stage of treatment. Glycemic alterations were found in 32.0% of the sample, with hyperglycemia being the most frequent change (28.2%). All patients had a GCS score ≤8, as this was an inclusion criterion, and 60.3% had pupillary changes.

**Table 3:** **Comparisons between patient conditions (dead or alive, independent or dependent) at 6 months after DAI in relation to characteristics at hospital admission**.

Characteristics related to admission	Survival		*p*-Value	GOS-E		*p*-Value
	No *n* (%)	Yes *n* (%)		Independent *n* (%)	Dependent *n* (%)	
**Sedation**
Yes	22 (91.7)	50 (92.6)	>0.999	41 (91.1)	6 (100.0)	>0.999
No	2 (8.3)	4 (7.4)		4 (8.9)	–	
**RR**	^a^	^a^		^c^	^c^	
Normal	19 (82.6)	45 (91.8)	0.257	38 (92.7)	5 (83.3)	0.432
Altered	4 (17.4)	4 (8.2)		3 (7.3)	1 (16.7)	
**Hypotension**
Yes	11 (45.8)	4 (7.4)	**<0.001**	4 (8.9)	–	>0.999
No	13 (54.2)	50 (92.6)		41 (91.1)	6 (100.0)	
**Bradycardia**
Yes	2 (8.3)	–	0.092	–	–	–
No	22 (91.7)	54 (100.0)		45 (100.0)	6 (100.0)	
**Tachycardia**
Yes	15 (62.5)	26 (48.1)	0.241	23 (51.1)	3 (50.0)	>0.999
No	9 (37.5)	28 (51.9)		22 (48.9)	3 (50.0)	
**Hypoxia by SpO_2_^b^**
Yes	9 (37.5)	3 (5.7)	**0.001**	3 (6.7)	–	>0.999
No	15 (62.5)	50 (94.7)		42 (93.3)	5 (100.0)	
**Hypoxia by PO_2_**	^c^	^c^		^e^	^e^	
Yes	3 (12.5)	8 (16.0)	>0.999	6 (14.3)	2 (33.3)	0.258
No	21 (87.5)	42 (84.0)		36 (85.7)	4 (66.7)	
**Hypoglycemia**	^d^	^d^		^c^	^c^	
Yes	1 (4.3)	2 (4.2)	>0.999	2 (4.9)	–	>0.999
No	22 (95.7)	46 (95.8)		39 (95.1)	6 (100.0)	
**Hyperglycemia**	^d^	^d^		^c^	^c^	
Yes	12 (52.2)	10 (20.8)	**0.008**	9 (22.0)	1 (16.7)	>0.999
No	11 (47.8)	38 (79.2)		32 (78.0)	5 (83.3)	
**Pupillary abnormalities**
Yes	21 (87.5)	26 (48.1)	**0.001**	21 (46.7)	4 (66.7)	0.419
No	3 (12.5)	28 (51.9)		24 (53.3)	2 (33.3)	

*HCFMUSP, 2013–2014*.

*^a^Excludes 6 cases without information*.

*^b^Excludes 1 case without information*.

*^c^Excludes 4 cases without information*.

*^d^Excludes 7 cases without information*.

*^e^Excludes 3 cases without information*.

*RR, respiratory rate; SpO_2_, peripheral oxygen saturation; PO_2_, partial pressure of oxygen*.

*Bold numbers correspond to significant p values*.

As shown in Table 4, almost all of the victims were hospitalized in the ICU (92.3%), with an average length of stay in this unit of 11.7 days (SD = 15.4) and a median of 7 days (range: less than 1–109 days). Of all patients, 69 (88.5%) were continuously sedated for an average of 4.1 days (SD = 4.3) and median of 3 days (ranging from less than 1–18 days). Fentanyl and propofol, used in 94.2 and 92.7% of sedated patients, respectively, were the most prescribed drugs for sedation. Most patients (61.5%) were treated with drugs that act on the CNS without the purpose of sedating. Among those, the most common drug types were anticonvulsants (83.3%), neuroleptics (70.8%), and benzodiazepines (25.0%).

**Table 4:** **Comparisons between patient conditions (dead or alive, independent or dependent) at 6 months after DAI in relation to characteristics related to hospital stay**.

Characteristics related to hospital stay	Survival		*p*-Value	GOS-E		*p*-Value
	No *n* (%)	Yes *n* (%)		Independent *n* (%)	Dependent *n* (%)	
**ICU stay**						
Yes	22 (91.7)	50 (92.6)	>0.999	42 (93.3)	6 (100.0)	>0.999
No	2 (8.3)	4 (7.4)		3 (6.7)	–	
**Use of continuous sedation**						
Yes	22 (91.7)	47 (87.0)	0.713	39 (86.7)	6 (100.0)	>0.999
No	2 (8.3)	7 (13.0)		6 (13.3)	–	
**Other treatments with drugs that act on the CNS**						
Yes	13 (54.2)	35 (64.8)	0.372	27 (60.0)	6 (100.0)	0.078
No	11 (45.8)	19 (35.2)		18 (40.0)	–	
**Surgery**						
Yes	13 (54.2)	29 (53.7)	0.970	23 (51.1)	5 (83.3)	0.204
No	11 (45.8)	25 (46.3)		22 (48.9)	1 (16.7)	
**Second surgery**						
Yes	4 (16.7)	11 (20.4)	>0.999	9 (20.0)	1 (16.7)	>0.999
No	20 (83.3)	43 (79.6)		36 (80.0)	5 (83.3)	
**Infection**						
Yes	7 (29.2)	13 (24.1)	0.634	8 (17.8)	4 (66.7)	**0.022**
No	17 (70.8)	41 (75.9)		37 (82.2)	2 (33.3)	
**Other complications**						
Yes	19 (79.2)	22 (40.7)	**0.002**	15 (33.3)	6 (100.0)	**0.003**
No	5 (20.8)	32 (59.3)		30 (66.7)	–	
**Early DAI signs in CT**						
Yes	22 (91.7)	33 (61.1)	**0.006**	25 (55.6)	6 (100.0)	0.070
No	2 (8.3)	21 (38.9)		20 (44.4)	–	
**ICP monitoring**						
Yes	2 (8.3)	1 (1.9)	0.223	–	1 (16.7)	0.118
No	22 (91.7)	53 (98.1)		45 (100.0)	5 (83.3)	
**ICH**						
Yes	11 (45.8)	8 (14.8)	**0.003**	4 (8.9)	4 (66.7)	**0.004**
No	13 (54.2)	46 (85.2)		41 (91.1)	2 (33.3)	
**Hypotension**						
Yes	7 (29.2)	6 (11.1)	0.096	5 (11.1)	1 (16.7)	0.548
No	17 (70.8)	48 (88.9)		40 (88.9)	5 (83.3)	
**Hypertension**						
Yes	15 (62.5)	38 (70.4)	0.601	31 (68.9)	6 (100.0)	0.170
No	9 (37.5)	16 (29.6)		14 (31.1)	–	
**Hypothermia**						
Yes	17 (70.8)	29 (53.7)	0.156	23 (51.1)	5 (83.3)	0.204
No	7 (29.2)	25 (46.3)		22 (48.9)	1 (16.7)	
**Hyperthermia**						
Yes	18 (75.0)	32 (59.3)	0.181	26 (57.8)	6 (100.0)	0.072
No	6 (25.0)	22 (40.7)		19 (42.2)	–	
**Hypoglycemia**						
Yes	7 (29.2)	16 (29.6)	0.967	13 (28.9)	3 (50.0)	0.363
No	17 (70.8)	38 (70.4)		32 (71.1)	3 (50.0)	
**Hypoglycemia in the first 5 days**						
Yes	5 (20.8)	7 (13.0)	0.498	7 (15.6)	–	0.578
No	19 (79.2)	47 (87.0)		38 (84.4)	6 (100.0)	
**Hyperglycemia**						
Yes	19 (79.2)	31 (57.4)	0.064	26 (57.8)	4 (66.7)	>0.999
No	5 (20.8)	23 (42.6)		19 (42.2)	2 (33.3)	
**Hyperglycemia in the first 5 days**						
Yes	13 (54.2)	25 (46.3)	0.521	21 (46.7)	4 (66.7)	0.419
No	11 (45.8)	29 (53.7)		24 (53.3)	2 (33.3)	

*HCFMUSP, 2013–2014*.

*ICU, intensive care unit; CNS, central nervous system; CT, computed tomography; ICP, intracranial pressure; ICH, intracranial hypertension*.

*Bold numbers correspond to significant p values*.

After hospital admission, DAI patients took on average 3.7 days (SD = 7.2) to achieve a score of 6 on the BMR item of the GCS. The median time was 1 day, ranging from less than 24 h to 32 days. However, at the 6-month follow-up, 23 patients (29.5%) did not reach a score of 6 on the BMR item of the GCS; 22 died before reaching that score, and 1 was in a persistent vegetative state until the end of the evaluation period.

As shown in Table 4, during hospitalization, most patients (53.8%) underwent surgery, and 19.2% had second surgery. Infections were recorded in 25.6% of cases and other complications in 52.6% of patients. Most of the victims showed early signs of DAI in the CT (70.5%), and ICH after DAI was detected in 24.2% of the patients, although ICP monitoring was performed in only three patients (3.8%).

In this study, the average hospital stay of patients was 19.1 days (SD = 22.9), and the median stay was 11 days (range: less than 1–111 days). Mortality at 6 months after DAI was 30.8%; among those who died, the average survival was 13.5 days (SD = 24.1), and the median survival was 4.5 days (range: less than 1–110 days). Most patients (63.0%) went home after discharge from IC/HCFMUSP; the others required hospitalization in other hospitals for continued care.

### Outcome at 6 Months

**Figure 1:**
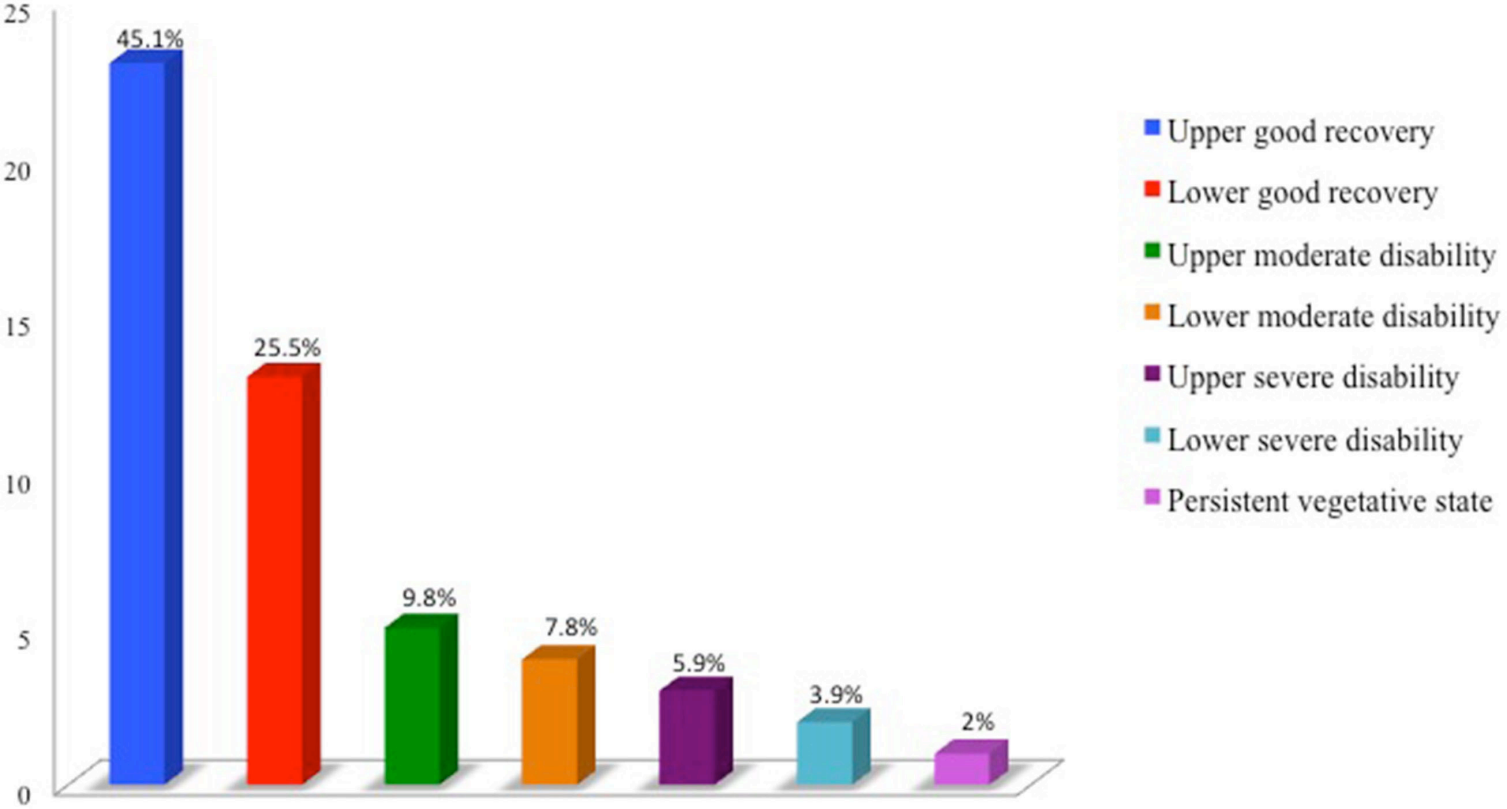
**DAI victims at 6 months after trauma: second functional capacity by the Extended Glasgow Outcome Scale (GOS-E)**. HCFMUSP, 2013–2014.

Six months after the DAI, according to the GOS-E, a large portion (45.1%) of these patients reached upper good recovery, and 25.5% showed lower good recovery. Individuals that were classified as “independent but disabled” amounted to 16.6% of the sample (9.8% upper moderate disability and 7.8% lower moderate disability); six subjects were classified as disabled and dependent (11.8%), with three ranked as having upper severe disability, two as lower severe disability, and one was in a persistent vegetative state. Thus, the vast majority of patients (88.2%) achieved recovery consistent with independent life at 6 months after DAI, while those severely disabled and dependent constituted a small proportion (11.8%; Figure 1).

### Factors Associated With Mortality

To identify factors associated with mortality within 6 months of the DAI, the 78 patients admitted to IC/HCFMUSP were grouped according to their vital state (dead or alive) in this period. In the analysis of the variables that reflected the severity of the trauma and TBI, as measured by the ISS, NISS, MAIS-Head, severity of DAI, and the presence of hypotension, statistically significant differences were observed for all variables (*p* < 0.001). Table 5 shows that death was the most frequent outcome among patients with severe DAI; out of 28 patients with severe DAI, 22 died (78.6%).

**Table 5:** **Comparisons between patient conditions (dead or alive, independent or dependent) at 6 months after DAI in relation to severity of DAI**.

DAI severity	Survival		*p*-Value	GOS-E		*p*-Value
	No *n* (%)	Yes *n* (%)		Independent *n* (%)	Dependent *n* (%)	
Mild/moderate	2 (8.3)	48 (88.9)	**<0.001**	44 (97.8)	1 (16.7)	**<0.001**
Severe	22 (91.7)	6 (11.1)		1 (2.2)	5 (83.3)	

*HCFMUSP, 2013–2014*.

*Bold numbers correspond to significant p values*.

In Table 3, a statistically significant difference is apparent between the groups (dead and alive) for the presence of hypotension (*p* < 0.001), hypoxia by SpO_2_ (*p* = 0.001), hyperglycemia (*p* = 0.008), and pupillary changes (*p* = 0.001). The values of the GCS assigned to DAI patients at admission were similar between the dead and alive groups (*p* < 0.137).

Table 4 shows that there was a statistically significant association between victims who died and those that presented, during hospitalization, with other complications (*p* = 0.002), early signs of DAI in CT (*p* = 0.006), and ICH (*p* = 0.003). The average values for GCS 48 h after the withdrawal of sedation were different between the dead and alive groups (*p* < 0.001).

The multiple logistic regression model tested the variables that achieved a *p*-value <0.05 in the analysis. The results point to the presence of hypoxia by SpO_2_ at admission (*p* = 0.029) and severe DAI (*p* < 0.001) as risk factors for death. No other variables reached significance during modeling and thus were not included in the model. The model in Table 6 was not a good fit for the data because the confidence intervals (CIs) of odds ratios (ORs; 95%) for both variables were large.

**Table 6:** **Logistic regression model of risk factors for mortality up to 6 months after DAI**.

Variable	*p*-Value	OR	CI for OR (95%)	
			Lower limit	Upper limit
Hypoxia by SpO_2_ at admission (yes)	**0.029**	18.77	1.36	259.27
DAI severe	**<0.001**	125.63	14.02	1,125.95

*HCFMUSP, 2013–2014*.

*OR, odds ratio; CI, confidence interval*.

*Bold numbers correspond to significant p values*.

To further explore the effects of factors other than DAI severity on the outcomes of this injury, a model excluding this variable was tested. This model showed that the presence of hypotension at admission (*p* = 0.016) and the value of NISS (*p* = < 0.001) were independently associated with mortality at the end of this regression (Table 7).

**Table 7:** **Logistic regression model of risk factors for mortality up to 6 months after DAI excluding severity of injury**.

Variable	*p*-Value	OR	CI for OR (95%)	
			Lower limit	Upper limit
Hypotension at admission (yes)	**0.016**	7.86	1.48	41.78
NISS	**<0.001**	1.14	1.07	1.22

*HCFMUSP, 2013–2014*.

*NISS, New Injury Severity Score*.

*Bold numbers correspond to significant p values*.

### Factors Related to Dependence 6 Months after DAI

Considering only the 51 patients who survived and participated in the 6-month follow-up, we identified two groups of patients with DAI: those needing assistance to carry out their daily activities (dependent) and those living independently 6 months after the trauma.

Analyzing the variables that reflect the severity of the trauma and of TBI, measured by the ISS (*p* = 0.003), NISS (*p* = 0.001), MAIS-Head (*p* < 0.001), ICH (*p* = 0.004; Table 4), infection (*p* = 0.022; Table 4), other complications (*p* = 0.003; Table 4), and severity of DAI (*p* < 0.001), all had shown statistically significant differences between the groups. Table 5 shows that patients with severe DAI were predominant in the dependent group, whereas among patients living independently, the injury was mild/moderate in almost all cases.

There was a statistically significant difference between the groups regarding the average length of ICU hospitalization (*p* < 0.001), continuous sedation (*p* = 0.001), and length of hospital stay (*p* = 0.037). Furthermore, the average value of GCS 48 h after withdrawal of sedation was different between the independent and dependent groups (*p* < 0.001). In comparing the two groups, the mean periods of time were higher in the dependent group, and the average GCS score was lower.

During modeling, the first variable entered in the model was severity of DAI (*p* ≤ 0.001), with which no other variable attained *p*-value <0.05. Similar to the mortality model, the CI of OR (95%) was quite broad for the result of regression to dependence (Table 8). Thus, once more, the severity of DAI was excluded from the model; in the final model (Table 9), only the duration of hospital stay was statistically significant in the logistic regression model with a *p*-value = 0.008.

**Table 8:** **Logistic regression model of risk factors for dependence at 6 months after DAI**.

Variable	*p*-Value	OR	CI for OR (95%)	
			Lower limit	Upper limit
DAI (severe)	**0.000**	205.00	11.02	3,813.02

*HCFMUSP, 2013–2014*.

*Bold numbers correspond to significant p values*.

**Table 9:** **Logistic regression model of risk factors for dependence at 6 months after DAI excluding severity of the injury**.

Variable	*p*-Value	OR	CI for OR (95%)	
			Lower limit	Upper limit
Duration of hospital stay (days)	**0.008**	1.07	1.02	1.12

*HCFMUSP, 2013–2014*.

*Bold numbers correspond to significant p values*.

## Discussion

Diffuse axonal injury is a microscopic lesion associated with significant mortality and morbidity. Evaluating the risk factors related to its consequences is of great importance for the implementation of appropriate multidisciplinary care and health policies aimed at the prevention and rehabilitation of patients with DAI.

Six months after DAI, 24 patients (30.8%) had died as a consequence of the trauma or of complications. Nevertheless, among those who survived this period (51 patients), 88.2% achieved GOS-E classification consistent with independent living, and 45.1% had shown full recovery from trauma, reporting a return to the pre-injury state. During this period, individuals with disabilities formed 29.4% of the sample, and 11.8% of those were dependent.

The literature reports worse outcomes – frequency of disability (40.0–87.5%) and dependency (20.0–41.3%) – for patients with DAI, evaluated by GOS or GOS-E at 6 months after injury (26–30). However, most participants in our study had mild (44.9%) or moderate (19.2%) DAI, and among these, only one (2.2%) was dependent during the period of the study (Table 5). Severe DAI stood out as a risk factor for mortality and dependence in this study, although the multivariate logistic regression analysis did not yield a good fit in the statistical models: CI of OR (95%) ranged from 13.65 to 395.89 for mortality and 11.2 to 3,813.02 for dependence. Though the confidence intervals clearly confirm the important role of the severity of DAI as a risk factor for adverse consequences of this injury, they were quite large because of the small number of deaths (two cases) and dependence events (one case) among patients with mild and moderate DAI, leading to instability in the model.

Diffuse axonal injury is difficult to diagnose in the acute phase. The combination of clinical signs and imaging may suggest the diagnosis, but confirmation is only possible postmortem (31). Using CT in the emergency room helps in the identification, diagnosis, and location of hemorrhages. Despite having low resolution in soft tissue evaluation after TBI, CT is considered a useful tool for identifying early signs of DAI in the acute phase and is widely employed in severe TBI victims because of the short duration of the exam, its wide availability in trauma centers, and its conduciveness for use in unstable patients (31).

Studies (3, 5, 11, 18, 27, 32) show that the frequency of DAI is higher in severe TBI victims who have indirect signs of injury on CT, such as intraventricular hemorrhage and subarachnoid hemorrhage, rather than a normal CT scan. In this study, for the analyses of association with the consequences of trauma, patients with normal CT scans were analyzed separately from those with signs suggestive of DAI, and the presence of these signs was more frequent among patients who progressed to death.

Studies that have emphasized imaging and biomarkers to estimate the severity and prognoses for patients with DAI corroborate the findings in this study: the greater the severity of DAI, the worse the outcome for the patient (32–34).

In addition to early signs of DAI in CT, other variables were associated with mortality: trauma severity indicators (ISS and NISS) and TBI (MAIS-Head); pupillary changes; hypotension; hypoxia measured by SpO_2_ and hyperglycemia on admission; GCS score after withdrawal of sedation; presence of ICH; and complications, other than infection, during hospitalization. Patients who died displayed these signs and clinical changes more often during hospital admission and hospitalization and had higher severity scores and lower GCS scores after withdrawal of sedation than those who survived.

Among trauma victim characteristics, hypoxia by SpO_2_ and hypotension on admission, in addition to the NISS, had significant effects in multivariate analysis. Nevertheless, the best fitting statistical model included only the last two variables and showed that patients with hypotension on admission were 7.86 times more likely to die than those without this symptom. Furthermore, each additional point in NISS value increased the chance of death in the first 6 months after DAI by 14.0%.

Physiological changes after TBI, such as hypoxia and hypotension, can result in secondary brain damage. Preserving the airways after trauma may result in favorable outcomes after severe TBI (13). Hypoxia and hypotension in severe TBI victims are common, and their occurrence in the initial hours is significantly associated with increased mortality (13, 35–37). Hypertension may relate to reduced brain perfusion and can worsen ICH and cerebral edema (36). Analysis of hypotension incidence in early resuscitation in TBI patients in the city of San Francisco showed that of 107 victims studied, 26 (24%) had hypotension, averaging 1.5 episodes per patient (mean duration 9.1 min). Of these patients with hypotension, 65.0% died, with the frequency of hypotension episodes directly proportional to the number of deaths (38).

Changes in glucose levels are either caused by metabolic or physiological disorders or are a stress response that reflects the severity of the injury, and they are related to unfavorable outcomes (39–41). Research (39) on 380 victims of TBI in the first 5 days of ICU admission indicated association of high glucose levels (≥160 mg/dL) in the first 24 h after admission with mortality and that mortality was higher in patients with hypoglycemia (<60 mg/dL).

Our results were broadly in line with a previous study of 78 patients diagnosed only with DAI; after an average of 12.3 months, factors significantly associated with mortality were hypotension, plasma glucose >144 mg/dL, low scores on the GCS at hospital admission, increased number of DAI lesions in CT and minor trauma injuries, presence of shock, coagulation disorders, transfusion, and no recovery of consciousness (5). In multivariate analysis, only the absence of recovery of consciousness and the large number of DAIs were identified as independent risk factors for mortality (5).

Studies that analyzed the association of TBI patient characteristics with mortality identified the following factors independently associated with mortality: the Marshall classification in CT (42), severity of the injury measured by CT (43), diffuse head injuries II–IV (44), lower score on the GCS (44, 45), hypotension (43–45), hyperglycemia (45, 46), hypothermia (46), SBP (43, 47), SpO_2_ (48), hypoxia (44, 45), shock (45), ICP monitoring (49), a score of 5 on MAIS-Head (47, 49), and a high score in the ISS (43, 45, 47, 49).

In this study, NISS was independently associated with mortality; however, no other reports of NISS application to groups of DAI patients were found. Although literature review indicates that NISS has better performance than ISS in predicting mortality (50), the scientific community is still cautious about replacing ISS with NISS for trauma severity identification, and thus, this indicator is underutilized.

In addition to the severity of the injury, dependence after 6 months following DAI was associated with higher scores in trauma severity indicators (ISS and NISS) and TBI (MAIS-Head), longer duration of sedation and hospitalization in the ICU and in the hospital, the presence of ICH, infection, other complications during hospitalization, and low scores on the GCS 48 h after the withdrawal of sedation.

Excluding the severity of DAI from dependence modeling, hospital stay was a significant predictor in the multiple regression model, with each additional hospitalization day increasing the chance of a patient being dependent 6 months after DAI by 7.0%.

A similar association was previously reported in an analysis of 41 patients with severe TBI in Hong Kong, which showed that prolonged hospitalization and advanced age were independent predictors of poor outcome 42 months after trauma (51). In another study (52) of 60 patients with severe TBI in the US, the length of the hospital stay and the length of the stay in the ICU were statistically associated with outcome 6 months after trauma. Moreover, in Brazil, a survey of patients with TBI conducted between 6 months and 3 years after trauma determined that individuals hospitalized for 12 days or more were 5.76 times more likely to become dependent than those with shorter hospital stays (24). According to Calvi et al. (33), factors associated with dependence or mortality in patients with TBI evaluated 3 months after trauma were lower GCS score on admission, higher ISS score, longer hospital stay, and longer stay in the ICU.

In a study (53) of 30 patients with DAI, severe TBI, pupillary abnormalities, higher ISS score, and lesions in the knee of the corpus callosum were associated with dependence 1 year after trauma. In multivariate analysis of these patients, only the corpus callosum lesions increased the risk of dependence 1 year after DAI. Assessment of patients with DAI 3 months after trauma in Japan showed that the lowest GCS score, the number of brain lesions identified on MRI, and the highest average value in ICP were significantly associated with dependence after trauma (54). In studies with patients with DAI and moderate TBI, greater severity of brain lesions identified by Marshall rating on CT (34) and higher number of DAIs in this exam (5) predicted dependence or mortality 6 months after trauma. However, the recovery of consciousness and lesions in the corpus callosum (5) were predictors of positive results after an average of 12 months after DAI. In addition, the assessment of factors related to dependence 6 months after trauma in 102 DAI patients showed that age, bilateral absence of pupillary reactivity to light, and multiple lesions observed in the corpus callosum and brainstem by MRI were associated with dependence (33).

Research on factors associated with DAI consequences typically uses imaging results for analysis (5, 34, 53, 54) and shows a relationship between changes in the images and consequences of DAI, although these results have been contradictory. In this study, only the presence of early signs of DAI in CT was tested as an independent variable, making it difficult to compare the findings.

The limitations of this study include the lack of MRI data, as the combination of imaging data with clinical findings might have made the diagnosis of DAI more reliable and allowed the comparison of clinical data with the brain lesions detected. Another limitation was the lack of available resources to perform this test on all participants; only 20 patients underwent this exam, and the difficulties of subjecting patients in a severe condition to this evaluation prevented considering MRI results in this study.

Applications of the results of this research must take into account some limitations. The sample included patients from a single institution, a referral center for the treatment of highly complex cases, which limits the generalization of the results. Moreover, the lack of hospital records on the patient’s clinical condition at the trauma scene and during transport limited the identification of pre-hospital stage factors that might be associated with the outcomes of DAI.

It should also be noted that the rehabilitation treatments of the patients were listed but not included in the association analyses, although it has been observed that all patients who were not independent at the last evaluation (6 patients) were seen by health-care experts to support their recovery.

## Conclusion

At 6 months after DAI, 24 patients (30.8%) died due to trauma or complications. However, among the survivors that were evaluated over this period (51 patients), 88.2% achieved GOS-E classification consistent with independent living, and 45.1% had full recovery from trauma. According to the GOS-E, six patients (11.8%) remained dependent at that point in time.

The sociodemographic characteristics of the study participants, related to trauma and PHC, were not associated with mortality and dependence. On the other hand, trauma severity indicators (ISS and NISS), TBI (MAIS-Head), and severity of DAI showed statistically significant associations with those consequences. Some clinical features observed at hospital admission were associated with mortality: pupillary changes, hypotension, hypoxia by SpO_2_, and hyperglycemia. During hospitalization, the GCS score after withdrawal of sedation, presence of ICH, and complications other than infection were associated with mortality and dependence. Early signs of DAI were associated only with mortality. Infection, continuous sedation time, and length of stay in the ICU and hospital were factors related to dependence.

Severe DAI stood out as a risk factor for mortality and dependence in the multivariate logistic regression analysis, although without good fit in the final model and with large CI of the OR (95%). Patients with severe DAI (*n* = 28) in almost all cases died or were dependent at 6 months after DAI. Along with severe DAI, the presence of hypoxia by SpO_2_ was a risk factor for mortality; however, variables of admission hypotension and NISS value showed the best fit for the model. Individuals who presented with hypotension on admission were 7.86 times more likely to die than victims without this change. The probability of dying was 14.0% higher for each additional point in the NISS.

Excluding the severity of DAI from dependence modeling, hospital stay was the variable that stood out in the multiple regression model, with each additional day of hospitalization increasing the chance of a patient remaining dependent 6 months after DAI by 7.0%.

## Author Contributions

RV contributed to the study design, manuscript development, and data analysis, wrote the manuscript, and performed the final review. WP contributed to data analysis and reviewed the manuscript. DO contributed to the writing and review of the manuscript. MT contributed to the manuscript review. AA contributed to the manuscript review. RS contributed to the study design, manuscript development, data analysis, review of the tables, writing of the manuscript, and the final review.

## Conflict of Interest Statement

The authors declare that the research was conducted in the absence of any commercial or financial relationships that could be construed as a potential conflict of interest.
